# Catalytic effect of high thermal conductive SiC on the kinetics and thermodynamics of vulcanization reaction of SBR/BR-filled nano-SiC

**DOI:** 10.1038/s41598-023-41337-5

**Published:** 2023-08-30

**Authors:** Sajad Rasouli, Amirreza Zabihi, Mohammad Fasihi

**Affiliations:** 1 School of Chemistry, Iran University of Science and Technology (IUST), P.O. Box 16844–13114, Tehran, Iran; 2 Compounding Laboratory, Department of Technology, Kian Tire Manufacturing Company, Tehran, Iran; 3 School of Chemical, Petroleum and Gas Engineering, Iran University of Science and Technology (IUST), P.O. Box 16844–13114, Tehran, Iran

**Keywords:** Polymers, Chemical engineering

## Abstract

Nano-silicon carbide (SiC) as a high thermal conductive material with an intrinsic thermal conductivity of ~ 490 W/m K was used to improve the cure characteristics, kinetics, and thermodynamics of curing reaction of styrene-butadiene rubber/butadiene rubber (SBR/BR) compounds. The considerations were carried out by non-isothermal differential scanning calorimetry (DSC). Results revealed that the presence of SiC shifted the peak and end temperatures of the curing peak to lower temperatures. The calculated activation energy of the curing reaction based on the Kissinger approach showed a descent from 409.8 to 93.8 kJ/mol by adding SiC from 0 to 7.5 phr (part per hundred rubber). Moreover, the obtained Gibbs free energy variation and equilibrium constant of the curing reaction proved that the reaction was absolutely forced and irreversible, which can be increasingly characterized as a one-way process. According to the results, SiC accelerated the curing reaction because of the increment of heat transfer into the compound. This phenomenon caused the increment of enthalpy variation of the vulcanization reaction, particularly at the SiC content of 5 phr. The achieved kinetic parameters via fitting an autocatalytic model based on the Sestàk–Berggren model by the Màlek method to describe the kinetics of the curing reaction indicated that the SiC filler had a catalytic effect on the curing reaction of SBR/BR-SiC, particularly after 2.5 phr of the filler.

## Introduction

As known, rubber is an example of elastomer type polymer with the ability to return to its original shape after being deformed or stretched^1, 2^. Rubbery materials have been used in a wide range of applications in the industrial and medical sections^3–6^. It seems to be unbelievable the imagination of the modern world without rubbers due to their crucial role in facilitating of human’s daily life.^7^. They have been used in numerous industries, from pharmaceutical^8^ and food packaging^9^ to tires on cars^10^ and so many other applications^11–13^. Factually, the popularity of the polymeric rubbers is due to their fantastic properties, *i.e.*, impermeability to both water and air^14^, excellent resistance to abrasion^15^, tearing and cutting over a wide range of temperature^16^, chemical resistance,^17^ and electrical insulation^18^. Tire manufacturing is one of the most well-known uses of rubbers in the car industry^19^. Approximately, forty to sixty percent of a tire is rubber^20^. Tread, under tread, crown, gum, inner layers, cover of beads are the main parts of a tire that usually are made of Styrene-butadiene-rubber (SBR), butadiene rubber (BR), Natural rubber (NR), polyisobutylene (PIB), etc.^21^. Despite the different rubbers in a tire, SBR and BR have allocated 60–70% of the whole rubbery parts to themselves^22^. This tremendous portion is due to their favorable properties, *i.e.*, hardness, chemical and water resistance, etc.^23^.

Generally, SBR and BR are blended in tire manufacturing to obtain novel collective properties that aren't achievable as individuals^24^. A high amount of the SBR loading content leads to better wet skid and traction properties. However, it results in a higher glass transition temperature (*T*_*g*_) with a negative impact on the tire performance at low temperatures^25^. In contrast, BR can reduce the *T*_*g*_ amount of a tire compound^26^. Accordingly, the *T*_*g*_ value of a typical tire composite can be controlled by adjusting the ratio of SBR/BR in the blend. In fact, the main reason for using the SBR/BR blend in the tire tread is to improve wet grip due to the presence of SBR and rolling resistance due to the presence of BR. Because the SBR has an ability to retain the composite grip on wet surfaces, while the BR part can resist against motion when a tire is on a surface^27, 28^.

Engineers have not used SBR/BR blends in a pure state. The blends have been turned into a composite to increase their efficiency for tire production. So far, few types of filler have been added to SBR/BR to achieve a rubbery composite with desirable properties^29–34^. Jin et al.^29^ investigated the influence of silica and SBR/BR blend ratio on the characteristics of filled tire tread compounds. Their considerations were dependent on the blend ratio, with the final goal to unmask the contribution of the SBR/BR ratio to the curing characteristics. They found that the maximum curing rate was reduced with increasing the SBR content, while the timespan reaching this rate was raised. This phenomenon occurred due to the polymer interactions with silica and the crosslinking reactivity. Xu et al.^30^ studied the dispersion of carbon black (CB)/silica in the SBR/BR composites and the compound properties in the presence of epoxidized NR as a compatibilizer. They proved that the crosslink density (CD) and the *T*_*g*_ value of the blend were increased when CB was replaced by silica. In another research work, Burgaz et al.^31^ considered the rheological and thermochemical properties of CB-reinforced NR/BR and NR/BR/SBR composites. Their results showed that the most optimized cure rate belonged to the NR/BR/SBR composite with highly linear BR. Moreover, they found that adding BR to the blend improved the CB dispersion due to suitable interactions between CB and the polymer chains.

So far, many research works have been performed concerning improving SBR/BR kinetic and thermodynamic properties, at the same time, the blend has great importance in the tire industry, especially tire tread. In contrast, there is a type of filler that can donate an extraordinary performance to the tire tread. For example, high thermal conductive materials have a unique property that can improve the curing characteristics of thermoset polymers^35^. Sanctuary et al.^36^ considered isothermal and non-isothermal cures for an epoxy resin-filled nano-alumina and cured with a triamine. They proved that with the increment of the filler content in the isothermal cure, the vitrification time was reduced because of increasing the heat flow, indicating an acceleration of the curing reaction by the filler. Recently, these fillers have attracted the attention of many scientists in the electrical, material, and computer sciences^37^. Examples of suitable high thermal conductive materials contain carbides; such as silicon carbide (SiC)^38^, nitrides; such as aluminum nitride (AlN) and boron nitride (BN)^39^, and oxides; such as alumina and silicon dioxide^40^. The high thermal conductive materials as filler can improve the thermal properties of SBR/BR-based compounds, particularly the kinetics of the curing reaction. The presence high thermal conductive reinforcement in the rubber composite improves the thermal diffusivity of the compound and can create a perfect heat transfer within a tire. Virtually, heat transfer in the cured composite (car tire) prevents the tire explosion while deriving due to the increment of the inside air temperature of the tire. In addition, these materials can decrease the required amount of energy for curing reactions, due to the perfect heat transfer. In the present work, SiC is used as a filler for SBR/BR blend as a high thermal conductive material with an intrinsic thermal conductivity of ~ 490 W/m K^40^. In this regard, different nano-SiC loading contents are applied to deeply study the influence of SiC on the cure characteristics, kinetics, and thermodynamics of the vulcanization reaction in the filled SBR/BR blend. To achieve this goal, first, kinetic parameters of an autocatalytic model are obtained to quantify the effect of SiC filler on the vulcanization reaction of the compound. According to Barghamadi et al.^41^, the calculation of activation energy of curing reaction can help to evaluate the impact of SiC on the required amount of energy for vulcanization. Then, the results of the kinetic measurement and the calculation of thermodynamic features of the curing reaction are used to unmask the optimum level of the filler for SBR/BR blend.

## Materials and methods

### Materials

Styrene-butadiene rubber (type 1500) with ~ 23.5 wt% styrene content, *T*_*g*_ value of − 40 °C and Mooney viscosity [ML_4+1_ at 100 °C] of ~ 52 MU was provided by Bandar Imam Petrochemical Co. (Khouzestan, Iran). High cis-BR (type 1210S) with a 97 wt.% cis content and Mooney viscosity [ML_4+1_ at 100 °C] of ~ 45 MU was purchased from Shazand Petrochemical Co. (Arak, Iran). Zinc oxide (ZnO), stearic acid, sulfur, and paraffin wax were bought from Arian Tootia Co. (Sharekord, Iran), Pars Pak Kimiya Co. (Tehran, Iran), Parto Googerd Asia Co. (Tehran, Iran) and Tosan Petro Energy Co. (Tehran, Iran), respectively. Antioxidant 4010NA (*N*-isopropyl-*N*′-phenyl-p-phenylenediamine) was purchased from Rongcheng Chemical General Factory Co., Ltd. (Shandong, China). The chemicals of Accelerator CZ (*N*-cyclohexyl-2-benzothiazolesulfenamide) and Accelerator DM (2,2'-dibenzothiazoledisulfde) were also supplied by Kemai Chemical Co., Ltd. (Tianjin, China). Nano-powder of SiC (type US2022) with a purity of 99%, particle size of 100 nm, bulk density of 3.216 g cm^−3^, and specific surface area of 25–50 m^2^/g, was provided by US Research Nanomaterials, Inc. (Florida, USA).

### Preparation of SBR/BR- filled SiC

In this work, the compound of SBR/BR incorporated with SiC (SBR/BR-SiC) was prepared in six steps: pre-blending, batch fusion, master-batch, resting, final, and forming steps. The pre-blending, master-batch, and final steps were performed by using a lab-scale internal mixer (Brabender Plasticorder, Netherlands). The batch fusion and forming steps were also accomplished on a lab-scale two-roll mill with a size of 6 × 12 in (Farrel Pomini, USA). The preparation process for SBR/BR-SiC is as follows:

(1) In the pre-blending step, the pure SBR and BR (with the blend ratio presented in Table 1) were added to the internal mixer. The fill factor was set to 75% of the maximum load capacity of the mixer. Then, the blend was mixed for 60 s at 50 rpm as the mastication process. In this step, the temperature control unit was fixed at 50 °C. Next, half of the SiC filler and paraffin wax were gently added to the blend (for 20 s) and mixed for 60 s with the rotor rate of 50 rpm. After a ram sweep for 30 s, and mixing for 60 s, the compound was discharged.

**Table 1:** Composition of SBR/BR-filled SiC compounds (in phr^a^).

Ingredients	Sample code			
	S0	S1	S2	S3
SBR	65.0^b^	65.0^b^	65.0^b^	65.0^b^
BR	35.0^b^	35.0^b^	35.0^b^	35.0^b^
Stearic acid	1.1	1.1	1.1	1.1
4010NA	2.0	2.0	2.0	2.0
ZnO	2	2	2	2
Sulfur	1.85	1.85	1.85	1.85
Paraffin wax	2.5	2.5	2.5	2.5
Accelerator CZ	1.0	1.0	1.0	1.0
Accelerator DM	0.5	0.5	0.5	0.5
SiC	0	2.5	5	7.5

^a^phr: Parts per hundred.

^b^The ratio of 65/35 was chosen for the blend of SBR/BR in the present research because the blend ratio is used in the tire industry to manufacture different compounds for various sections of a car tire, *i.e.*, tread and side wall., particularly in Kian Tire Company (one of the biggest tire Company in Iran).

1. In the batch fusion step, the pre-blended compound was milled on the two-roll mill for 6 min. Then, the mixture was discharged and prepared for the master-batch step.
2. In the master-batch step, the sheet compound was poured into the mixer and mixed for 60 s under 50 rpm. After 60 s, the rest of the SiC filler and paraffin wax were added to the compound—similar to the conditions of the pre-blending step—and mixed for another 60 s. The temperature control unit was also set at 50 °C. After twice ram sweeps for 30 s and mixing for 60 s, the compound was discharged.
3. In the resting step, the master-batch was led to relax in the ambient temperature for 24 h.
4. In the final step, the rested master-batch was poured into the mixer and mixed for 60 s under 50 rpm. Then, the remaining chemicals, *i.e.*, ZnO, antioxidant 4010NA, stearic acid, sulfur, Accelerator CA, and DM (according to Table 1), were slowly added to the compound for 30 s. Next, the mixture was mixed for 60 s. After twice ram sweeps for 30 s and mixing for 50 s, the final compound was discharged from the mixer.
5. In the forming step, the final compound was milled on the two-roll mill and formed for 4 min. In the end, the sheet compound was discharged from the two-roll mill. The final thickness of the uncured sheet is 9 ± 1 mm.

It should be mentioned that the two-roll mill apparatus was equipped with water circulation (with a temperature of 30 °C) for cooling. Also, the maximum temperature at the mixing procedure in the internal mixer at the master-batch step was 120 °C. In the master-batch step, the compound has no curing agent (sulfur); hence the temperature of 120 °C would not create any problem. Moreover, the temperature control unit was set at 50 °C in the master-batch step, and the temperature of 120 °C was just momentary. Factually, the temperature is raised in the internal mixer at the master-batch stage due to the friction between the polymer chains. In the present work, the preparation process as mentioned above, was used for all the compounds of SBR/BR-SiC with the SiC amounts of 0–7.5 phr (parts per hundred) (see Table 1).

### Testing performed on SBR/BR-SiC

#### Differential scanning calorimetry (DSC)

The cure characteristics of the SBR/BR-SiC compounds were studied using a Mettler Toledo DSC 822 thermal analyzer (Switzerland). Samples of the compounds were inserted into the apparatus, and the DSC scan was implemented from 25 °C (room temperature) to 300 °C with three heating rates of 10, 15, and 20 K/min. The scanning process was performed in the nitrogen atmosphere, and the mass of each sample was ~ 1.5 mg. Before the DSC analysis, zinc as the metal standard was used to calculate the cell constant for calibration of the device.

### Theoretical background of statistical analysis

#### Kinetics of vulcanization reaction of SBR/BR-SiC

Using a DSC scan, the kinetics of the curing reaction can be quantified by characterizing the heat signal generated during the vulcanization reaction of SBR/BR-SiC as the functions of time and temperature. The area under the heat flow-time (*H-t*) curve in DSC is expected to be the reaction conversion, which is defined via the following equation^42^:

1 $$\alpha =\frac{H(t)}{{H}_{T}}$$

In which *α* indicates the extent of the vulcanization reaction. The parameters of *H*(*t*) and *H*_*T*_ are the heat flow within time *t* and the total required heat flow for the SBR/BR-SiC vulcanization, respectively. The heat evolution in the *H-t* curve of DSC is assumed to be proportional to the consumption of the reactive groups.

The vulcanization reaction rate during the curing process is defined via the following equation^43^:

2 $$\frac{d\alpha }{dt}=(\frac{1}{{H}_{T}})\frac{d H(t)}{dt}$$

where *dα*/*dt* and *dH*(*t*)*/dt* are the vulcanization and heat flow rates, respectively. In general, most vulcanization kinetics are defined via the following equation ^44^:

3 $$\frac{d\alpha }{dt}=A exp(\frac{{-E}_{a}}{RT})f(\alpha )$$

where *A* and *E*_*a*_ are the pre-exponential factor and activation energy, respectively. Also, the well-known parameters of *R* (8.3144 J/mol K) and *T* are the gas constant and absolute temperature, respectively. The function of *f*(*α*) presents the kinetic model as well^45^. According to the classical kinetics of chemical reactions, *f*(*α*) supposes that the reaction rate is proportionate to the non-reaction substance as follows^46^:

4 $$f\left(\alpha \right)={(1-\alpha )}^{n}$$

where *n* illustrates the reaction order. Moreover, the autocatalytic kinetic model is used to identify the form of an additional complication of the kinetics of a chemical reaction. This type of kinetic model assumes that the reaction obeys the Sestàk–Berggren equation^47^, which is written by the following equation:

5 $$f\left(\alpha \right)={\alpha }^{m}{(1-\alpha )}^{n}$$

In which the parameter of *m* indicates the order of autocatalytic reaction. The Sesták–Berggren equation representing a powerful tool for the description of kinetic data by the model-fitting methods. Also, it has the ability to describe a variety of kinetic data of organic origin, particularly rubbers. This kinetic equation enables us to describe the kinetics of complex processes without a deeper insight into their mechanism^47^. Moreover, the Sesták–Berggren equation has been widely used in the investigation of not only isothermal, but also non-isothermal processes.

With the combination of the equations above, and the definition of heating rate in the DSC analysis (*β* = *dT/dt*^44^), the general kinetic model in the non-isothermal conditions can be described via the following equation:

6 $$\frac{d\alpha }{dT}=\frac{A}{\beta } exp(\frac{{-E}_{a}}{RT}){\alpha }^{m}{(1-\alpha )}^{n}$$

In this work, the statistical analysis related to the kinetics of vulcanization of SBR/BR-SiC is carried out using the Python programming language^44^.

#### Thermodynamics of vulcanization reaction of SBR/BR-SiC

In this work, to calculate the change in Gibbs free energy ($\Delta G$) of the curing reaction of SBR/BR-SiC, Eyring–Polanyi equation ^48^ is used as follows:

7 $$k=\frac{{k}_{B}\cdot T}{{h}_{p}}exp(-\frac{\Delta G}{RT})$$

In which *k*_*B*_ and *h*_*p*_ are the Boltzmann and Planck constants with the values of 1.3806 × 10^−23^ J/K and 6.6261 × 10^−34^ J/s, respectively. Moreover, *k* is the rate constant which is defined by the Arrhenius equation^49^ as follows:

8 $$k=A exp(-{E}_{a}/RT)$$

Furthermore, the equilibrium constant (*K*) is also calculated using the isotherm of vant Hoff^50, 51^ as follows:

9 $$\Delta G=-RT\cdot lnK$$

In this work, statistical analysis is performed using the Python programming language^44^.

## Results and discussion

### Cure characteristics of SBR/BR-SiC

**Figure 1:**
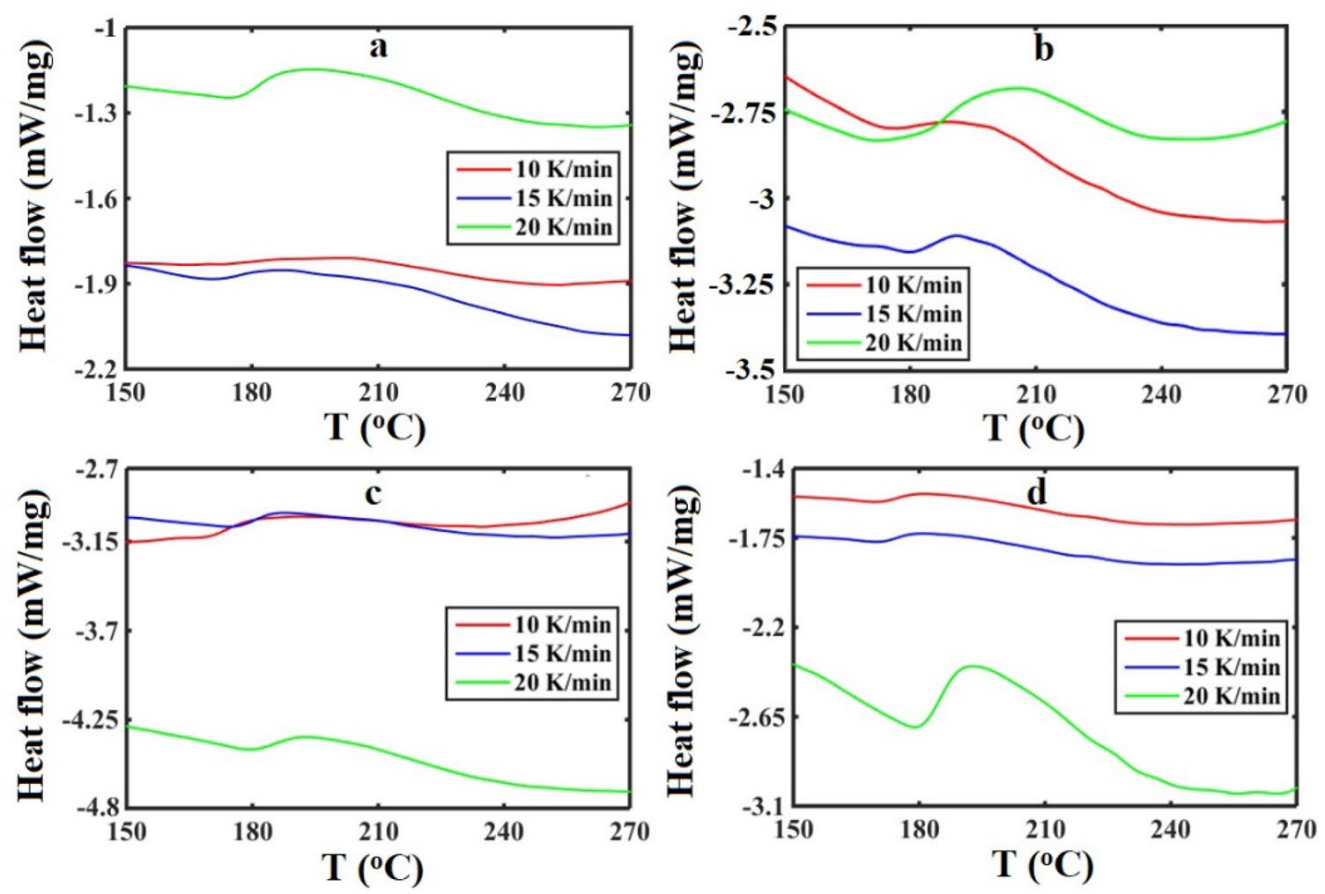
The local peak of DSC curve illustrating the vulcanization peak of the SBR/BR-SiC samples with the filler contents: (**a**) 0, (**b**) 2.5, (**c**) 5, and (**d**) 7.5 phr, at different heating rates of 10, 15 and 20 K/min.

DSC curves of the SBR/BR-SiC samples, with the SiC contents of 0, 2.5, 5, and 7.5 phr, at different heating rates of 10, 15, and 20 K/min were shown in Fig. S1 (Supporting Information). Specifically, local peaks of the DSC curves indicating the vulcanization of SBR/BR-SiC samples – at the temperature range of 150 K ≤ *T* ≤ 270 K—are exhibited in Fig. 1.

**Figure 2:**
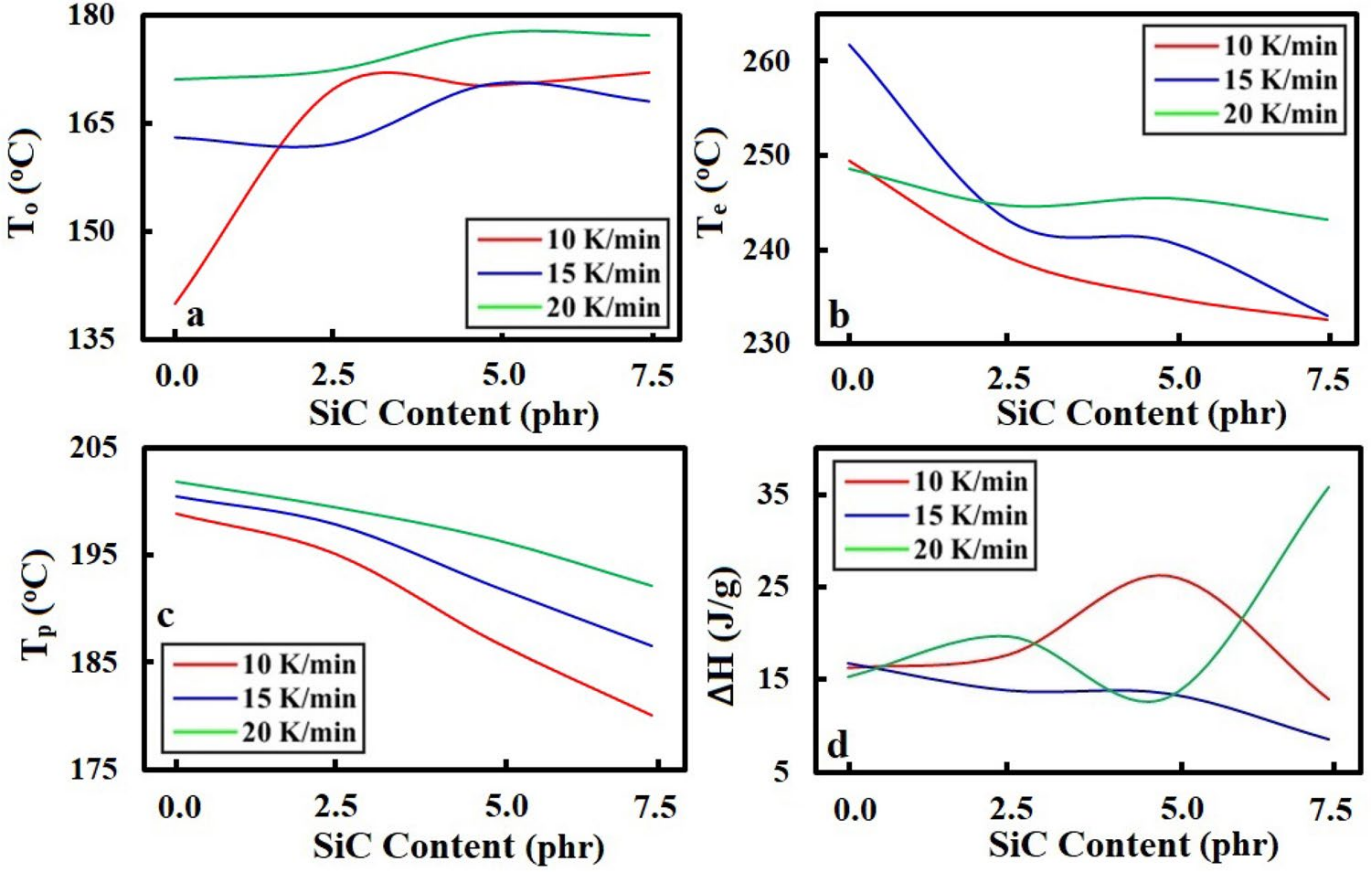
Cure characteristics: (**a**) onset temperature (*T*_*o*_), (**b**) end temperature (*T*_*e*_), (**c**) maximum temperature (peak temperature) (*T*_*p*_), and (**d**) enthalpy variation (*∆H*) of the vulcanization peak versus the SiC content (in phr) at different heating rates of 10, 15 and 20 K/min.

The cure characteristics, including the onset (*T*_*o*_), end (*T*_*e*_) and maximum (or peak) (*T*_*p*_) temperatures, as well as the enthalpy variation (*∆H*) of the vulcanization peak for all the samples were extracted from Fig. 1, and then listed in Table S1. These characteristics were calculated according to Raveshtian et al.^44^. In order to create deep insight into the trend of changes of the obtained cure characteristics, the parameters of *T*_*o*_, *T*_*e*_, *T*_*p*_, and *∆H* are presented as a figure in Fig. 2.

As seen in Fig. 2, the onset temperature was generally increased with increasing the SiC content, while the end and peak temperatures were reduced with the loading content of the filler. These results indicate that high thermal conductive SiC in the SBR/BR blend leads to moving the maximum curing and the completion of the vulcanization procedure to the lower temperatures. This issue helps to increase the rate of the vulcanization reaction in the SBR/BR-SiC compound, which saves more energy in the tire industry. As seen in Fig. 2d, the enthalpy variation of the curing reaction of SBR/BR containing 5.0 phr SiC—at 10 K/min—was become 1.6 times than the pure blend, which means the filler helped to further vulcanization into the compound. Because the higher value of *∆H* was created due to more reactions between sulfurs and the polymer chains, this phenomenon makes further cross-links between the polymer chains by sulfur bonds. Whereas increasing the SiC content to more than 5.0 phr led to a change in the trend and a reduction of the enthalpy variation. The results of our previous study on the NR-filled Al_2_O_3_^44^ have proved that the aggregation of filler particles plays a crucial role in reducing of the enthalpy variation after a certain amount of the filler content. Accordingly, the aggregation decreased the SiC performance because the surface area of the filler particles was reduced by sticking the filler particles together. Therefore, the aggregation had a worse impact on the vulcanization of the SBR/BR-SiC compound after 5.0 phr of the SiC filler. It should be noted that the DSC analysis with the heating rate of 10 K/min was just chosen to compare the curing enthalpy variation of the samples with different amounts of SiC because this value is so reliable for DSC of rubbery compounds^44, 52, 53^.

It can be seen from the obtained results of the cure characteristics that high thermal conductive SiC has a favorable impact on the curing process of the SBR/BR-SiC blend. Because the SiC particles—due to their ability to accelerate the heat transfer—can play a positive role in the perfect heat distribution into the compound, which can speed up the vulcanization process.

### Kinetics of curing reaction of SBR/BR-SiC

#### Conversion of curing reaction

**Figure 3:**
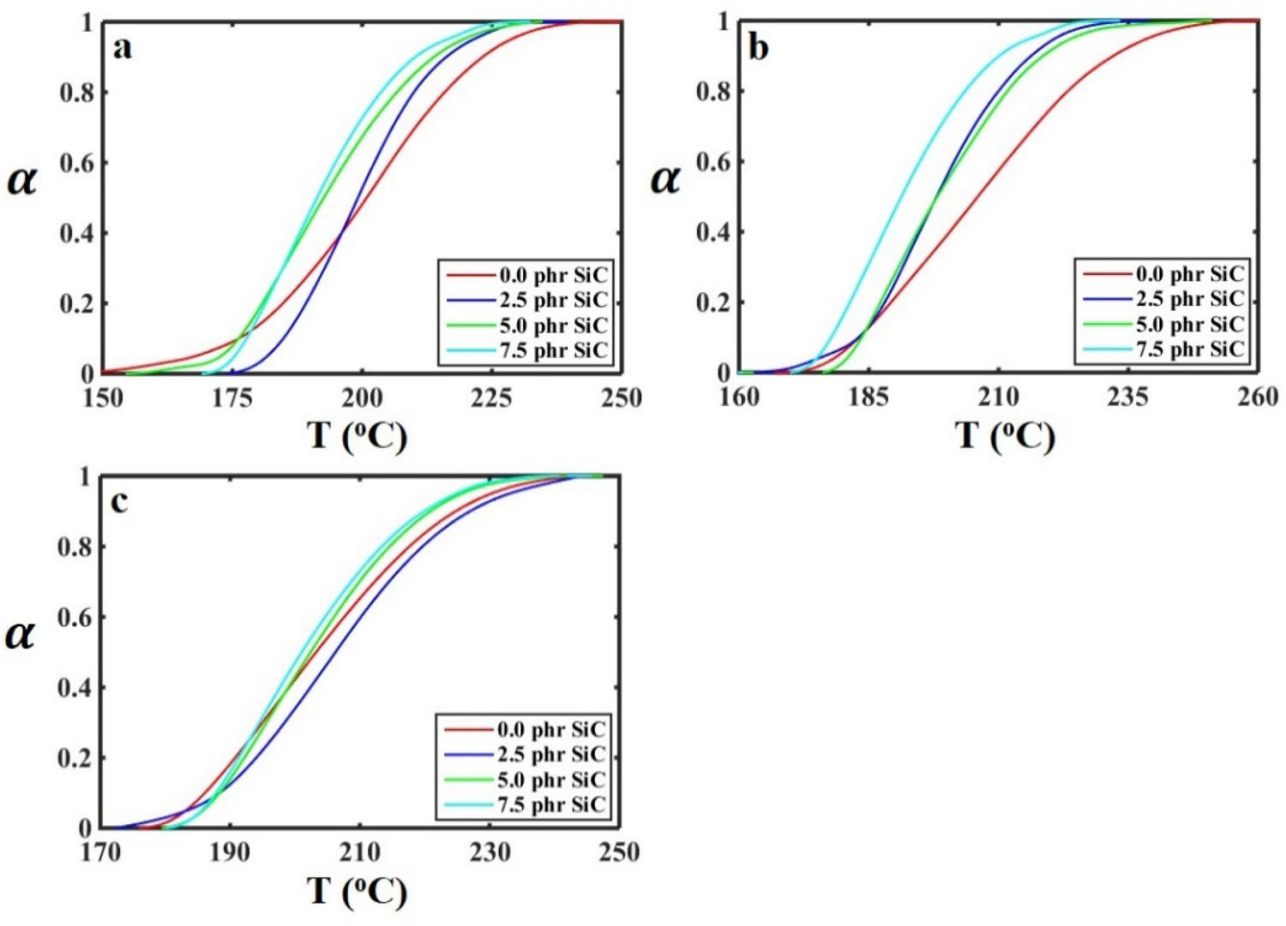
Conversion of curing reaction ($\alpha$) as a function of temperature for the SBR/BR-SiC samples with different contents of SiC at the heating rates: (**a**) 10 K/min, (**b**) 15 K/min, and (**c**) 20 K/min.

For more clarification regarding the effect of SiC on the curing properties of the SBR/BR-SiC compound, the conversion of the curing reaction should be considered. In this research work, the conversion of curing reaction ($\alpha$) during the vulcanization peak was calculated for each sample by Eq. (1). The conversion results concerning to the temperature at different heating rates and the SiC contents are shown in Fig. 3.

As seen in Fig. 3, The SiC filler enhanced the conversion of vulcanization reaction in the SBR/BR-SiC samples even before the peak temperature. Moreover, it helped to complete the curing process at lower temperatures. This behavior was seen for all the samples at different heating rates of 10, 15, and 20 K/min. Hence, the SiC particles accelerated the curing reaction of SBR/BR-SiC because of increasing the heat distribution within the bulk of the compound. This phenomenon can be expressed by the catalytic effect of SiC on the vulcanization reaction of the compounds. In addition, the conversion-temperature curves presented in Fig. 3 can be used as primary data for kinetic calculation of the vulcanization of the SBR/BR-SiC compounds.

#### Activation energy of curing reaction

The minimum amount of required energy for reactants to result in a chemical reaction is defined as activation energy^54^. In conjunction with the rubber compounds, activation energy is commonly estimated based on the Kissinger approach^55^. Accordingly, the Kissinger approach is used to calculate the activation energy of the curing reaction of the SBR/BR-SiC compounds in the present work. In the Kissinger method, overall activation energy can be quantified via the following equation^55^:

10 $$\mathit{ln}\left(\frac{\beta }{{T}_{p}^{2}}\right)=\mathit{ln}\left(-\frac{AR}{{E}_{a}}{\left[\frac{df(\alpha )}{d\alpha }\right]}_{{T}_{p}}\right)-\frac{{E}_{a}}{{R T}_{p}}$$

**Figure 4:**
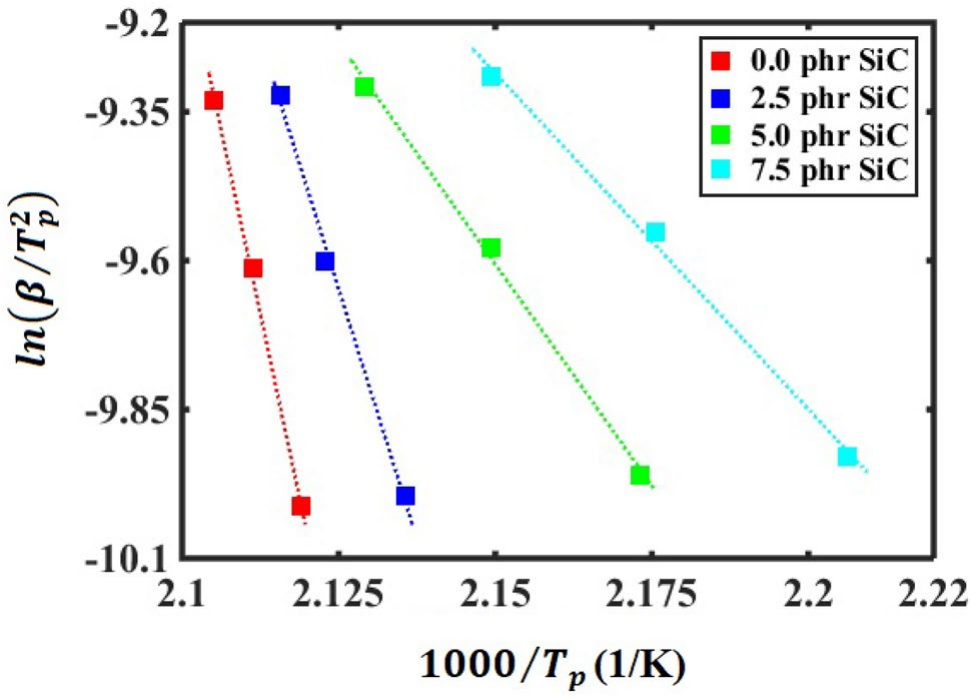
Kissinger plot of $\mathit{ln}\left(\beta /{T}_{p}^{2}\right)$ vs. $1000/{T}_{p}$ for the SBR/BR-SiC compounds with 0, 2.5, 5, and 7.5 phr of the filler. The dashed lines are the fitted linear equations on the results, with the *R*-squared values of *R*^2^ = 0.99.

In a non-isothermal curing process, the activation energy, pre-exponential factor, heating rate, and peak temperature are related together via Eq. (10). According to the above equation, the activation energy can be calculated as the slope of the Kissinger plot of $\mathit{ln}\left(\beta /{T}_{p}^{2}\right)$ −$1/{T}_{p}$. Figure 4 shows the Kissinger plot of $\mathit{ln}\left(\beta /{T}_{p}^{2}\right)$ vs. $1000/{T}_{p}$.

As shown in Fig. 4, the three square points for each compound correspond to the heating rates of 10, 15, and 20 K/min, and the dashed line is the fitted linear equation to find the slope of the figure. The linear fits on the results of Fig. 4 are the best-fitted linear equations with the *R*-squared of 0.99. The excellent linear fitting of the data indicates that the results fit the Kissinger model perfectly. Therefore, overall activation energy was calculated at 409.8, 279.2, 123.7, and 93.8 kJ/mol for the SBR/BR-SiC compounds containing 0, 2.5, 5, and 7.5 phr of the filler, respectively. The obtained activation energies illustrate that adding high thermal conductive SiC to the SBR/BR-SiC compound decreases the activation energy barrier of the compound during the curing process. This means the SiC filler makes the occurrence of vulcanization reaction easier.

#### Kinetic model of curing reaction

In this work, Màlek method^56^ is applied to quantify the kinetic model of the vulcanization reaction of SBR/BR-SiC. In this method, first, activation energy is determined, then two characteristic functions of $\gamma (\alpha )$ and $\varphi (\alpha )$ are defined based on the reaction rate and activation energy to find the kinetic model as follows^57^:

11 $$\gamma \left(\alpha \right)=(\frac{d\alpha }{dT})\beta {e}^{{E}_{a}/RT}$$

12 $$\varphi \left(\alpha \right)=(\frac{d\alpha }{dT})T\omega ({E}_{a}/RT)$$

The term $\omega ({E}_{a}/RT)$ in Eq. (12) is the function of temperature integrals whose values can be estimated with a fourth-order rational explanation of Chen et al.^57^ through the following equation:

13 $$\omega \left({E}_{a}/RT\right)=\frac{{\left({E}_{a}/RT\right)}^{3}+18{\left({E}_{a}/RT\right)}^{2}+88\left({E}_{a}/RT\right)+96}{{\left({E}_{a}/RT\right)}^{4}+20{\left({E}_{a}/RT\right)}^{3}+120{\left({E}_{a}/RT\right)}^{2}+240\left({E}_{a}/RT\right)+120}$$

In addition, to find the kinetic model and its parameters of *n* and *m*, the characteristic functions are normalized within (0,1) intervals as follows:

14 $${\gamma }_{norm}\left(\alpha \right)=\frac{\gamma \left(\alpha \right)}{\mathrm{max}[\gamma \left(\alpha \right)]}$$

15 $${\varphi }_{norm}\left(\alpha \right)=\frac{\varphi \left(\alpha \right)}{\mathrm{max}[\varphi \left(\alpha \right)]}$$

**Figure 5:**
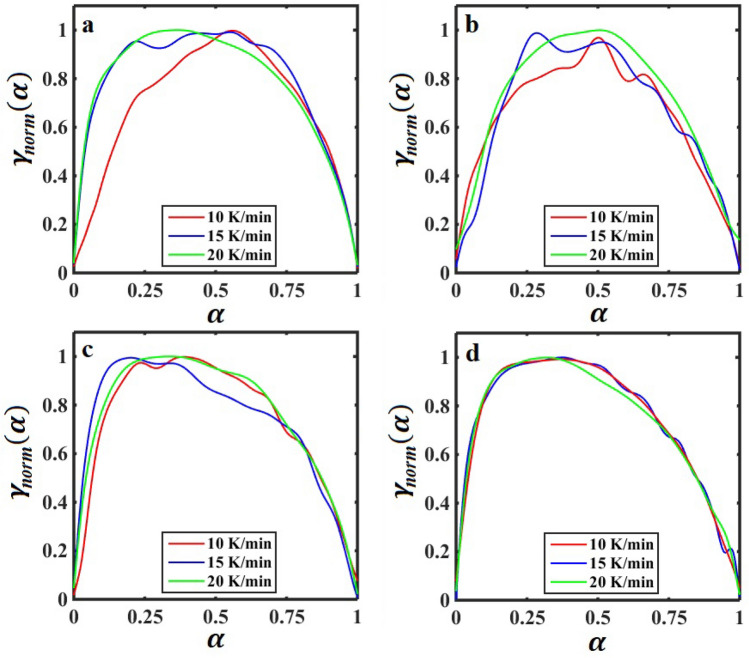
The normalized characteristic function of ${\gamma }_{norm}\left(\alpha \right)$ versus $\alpha$ (conversion of curing reaction) for the SBR/BR-SiC compound with the filler contents: (**a**) 0, (**b**) 2.5, (**c**) 5, and (**d**) 7.5 phr at different heating rates of 10, 15, and 20 K/min.

**Figure 6:**
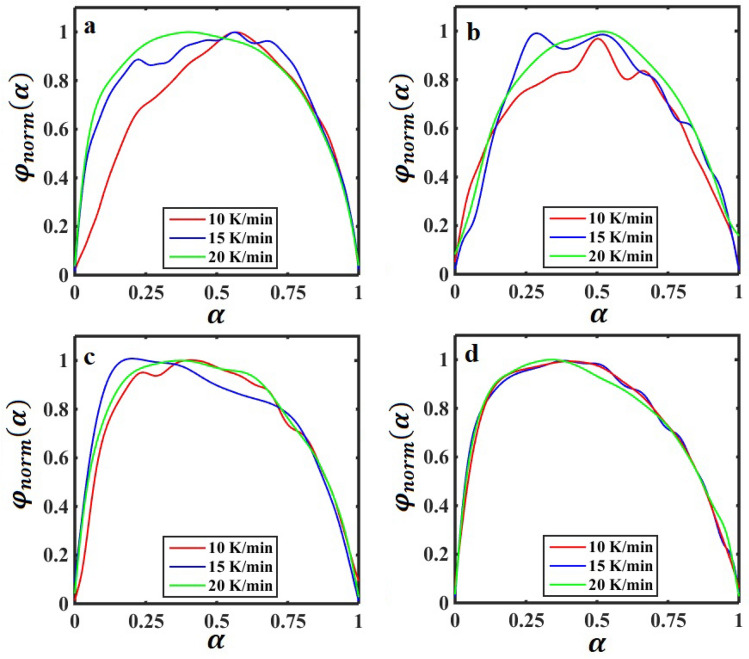
The normalized characteristic function of ${\varphi }_{norm}\left(\alpha \right)$ versus $\alpha$ (conversion of curing reaction) for the SBR/BR-SiC compound with the filler contents: (**a**) 0, (**b**) 2.5, (**c**) 5, and (**d**) 7.5 phr at different heating rates of 10, 15, and 20 K/min.

The calculated normalized characteristic functions of ${\gamma }_{norm}\left(\alpha \right)$ and ${\varphi }_{norm}\left(\alpha \right)$ versus $\alpha$ are illustrated in Figs. 5 and 6, respectively. As seen in Figs. 5 and 6, the curves exhibit maxima at ${\alpha }_{m}^{\gamma }$ and ${\alpha }_{m}^{\varphi }$, respectively. According to the maxima in Figs. 5 and 6, the kinetic parameters of *n* and *m* can be calculated, and subsequently, the most appropriate kinetic model can be determined. The values of ${\alpha }_{m}^{\gamma }$ and ${\alpha }_{m}^{\varphi }$ are listed in Table 2.

**Table 2:** The values of ${\alpha }_{m}^{\gamma }$ and ${\alpha }_{m}^{\varphi }$ (the location of maximum in the ${\gamma }_{norm}\left(\alpha \right)$-$\alpha$ and ${\varphi }_{norm}\left(\alpha \right)$-$\alpha$ curves, respectively) are extracted from Figs. 5 and 6, respectively. The value of *q* is equal to ${\alpha }_{m}^{\gamma }/(1-{\alpha }_{m}^{\gamma })$.

Sample code	$\beta$(K/min)	${\alpha }_{m}^{\gamma }$	${\alpha }_{m}^{\varphi }$	*q*
S0	10	0.536	0.57	1.157
	15	0.423	0.57	0.733
	20	0.391	0.41	0.643
	Mean	0.450 ± 0.06	0.516 ± 0.07	0.844 ± 0.22
S1	10	0.488	0.51	0.955
	15	0.299	0.29	0.428
	20	0.464	0.52	0.866
	Mean	0.417 ± 0.08	0.440 ± 0.10	0.750 ± 0.23
S2	10	0.439	0.40	0.783
	15	0.258	0.20	0.348
	20	0.390	0.37	0.639
	Mean	0.362 ± 0.07	0.323 ± 0.08	0.590 ± 0.18
S3	10	0.368	0.36	0.583
	15	0.364	0.37	0.574
	20	0.361	0.34	0.565
	Mean	0.364 ± 0.002	0.356 ± 0.01	0.574 ± 0.007

According to Màlek^57^, the shape of the curves presented in Figs. 5 and 6 indicate that the vulcanization process of SBR/BR-SiC can be described by the two-parameter autocatalytic kinetic model (see Eq. 6). In the Màlek method, the kinetic parameters of *n* and *m* are related together by the following equation^56^:

16 $$q=\frac{m}{n}=\frac{{\alpha }_{m}^{\gamma }}{1-{\alpha }_{m}^{\gamma }}$$

The *q* values were obtained by using Table 2 and Eq. (16), and the results are summarized in Table 3. To calculate the parameters of *n* and *m*, first, the parameter of *n* should be calculated by computing the slope of the following equation (from the combination of Eqs. 6 and 16)^56^:

**Table 3:** The kinetic parameters of *m*, *n*, *A* (pre-exponential factor) and *E*_*a*_ (activation energy) for the Sestàk–Berggren model by the Màlek method of the samples.

Sample code	$\beta$(K/min)	*m*	*n*	*A* (*1/min*)	*E*_*a*_ (*kJ/mol*)
S0	10	0.845	0.730	0.713	409.8
	15	0.454	0.618	0.668	
	20	0.414	0.644	1.124	
S1	10	0.670	0.701	0.884	279.2
	15	0.421	0.984	1.628	
	20	0.709	0.818	1.617	
S2	10	0.644	0.821	0.749	123.7
	15	0.318	0.913	0.911	
	20	0.454	0.709	1.364	
S3	10	0.450	0.771	0.715	93.8
	15	0.441	0.767	1.067	
	20	0.412	0.729	1.362	

17 $$ln\left[(d\alpha /dT)\beta exp({E}_{a}/RT)\right]=lnA+n ln\left[{\alpha }^{q}(1-\alpha )\right]$$

Next, the parameter of *m* can be calculated using Eq. (16). As seen in the above equation, the intercept of the plot of $ln\left[(d\alpha /dT)\beta exp({E}_{a}/RT)\right]$ vs. $ln\left[{\alpha }^{q}(1-\alpha )\right]$ results in *lnA*. The calculated parameters of *n, m,* and* A* for all the samples are listed in Table 3. Additionally, the obtained kinetic model with its kinetic parameters for each SBR/BR-SiC compound is also presented in Table 3.

**Figure 7:**
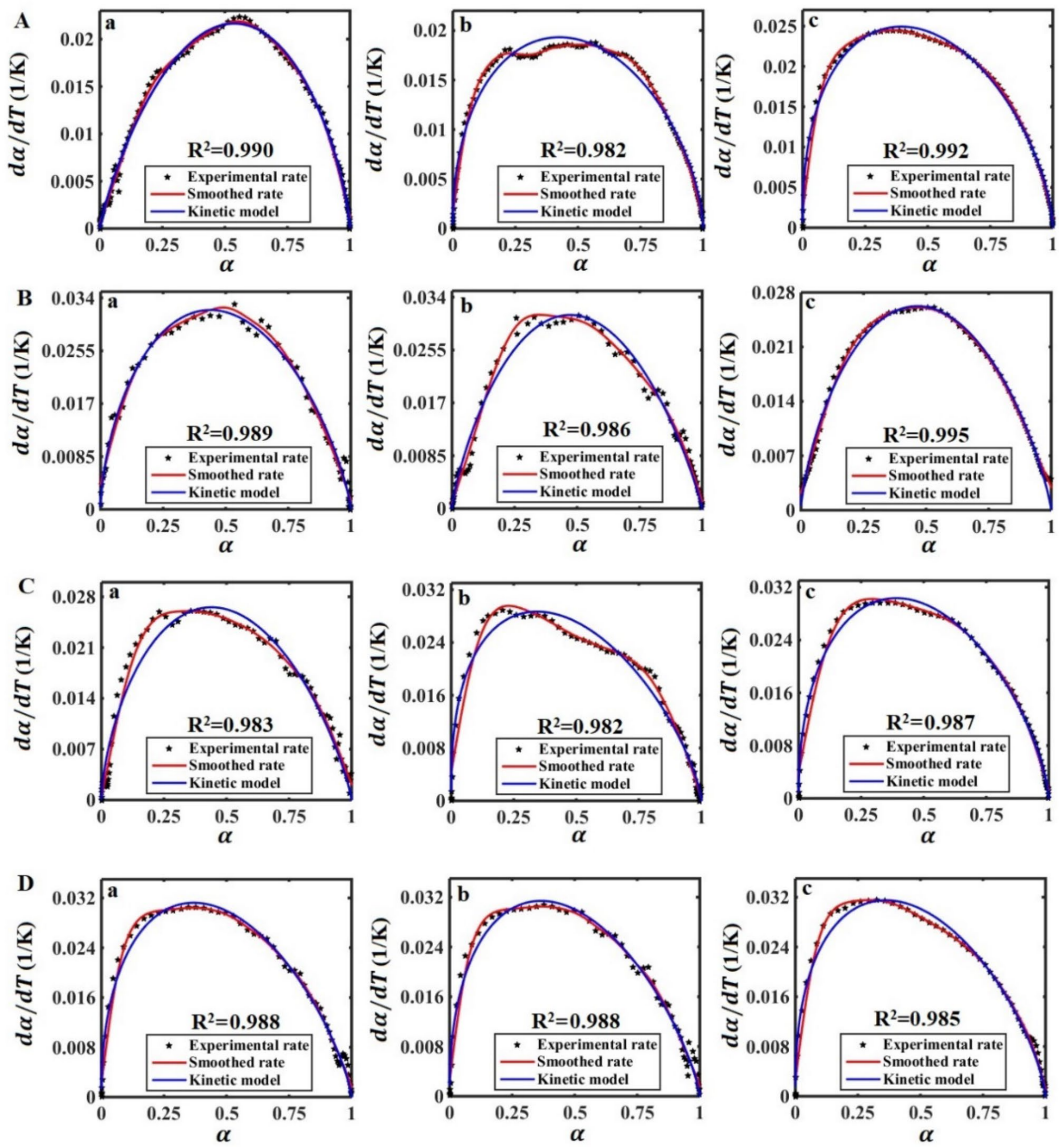
Comparison of experimental values of the vulcanization reaction rate (*d *$\alpha$*/dT*) and the predicted amounts via the applied kinetic model for the compounds with (**A**) 0, (**B**) 2.5, (**C**) 5, and (**D**) 7.5 phr of the SiC filler at different heating rates of (a) 10, (b) 15 and (c) 20 K/min.

Figure 7 shows the experimental values of the vulcanization reaction rate of the SBR/BR-SiC compounds with the predicted values by the obtained kinetic model. The figure indicates that the Sestàk–Berggren model deduced by Màlek method can expect the entire process of the vulcanization reaction of SBR/BR-SiC. The results reported in Table 3 disclose that the addition of high thermal conductive SiC to the SBR/BR compound leads to a change in the kinetic parameters of the vulcanization reaction. This means that the SiC filler causes a significant impact on the curing reaction of the compound in the vulcanization process. Therefore, the kinetic parameters obtained in this research work can be used to describe the curing kinetics of SBR/BR-SiC in the tire industry to achieve the particular degree at a certain time and temperature. The presented kinetic parameters are very important from the practical point of view.

**Figure 8:**
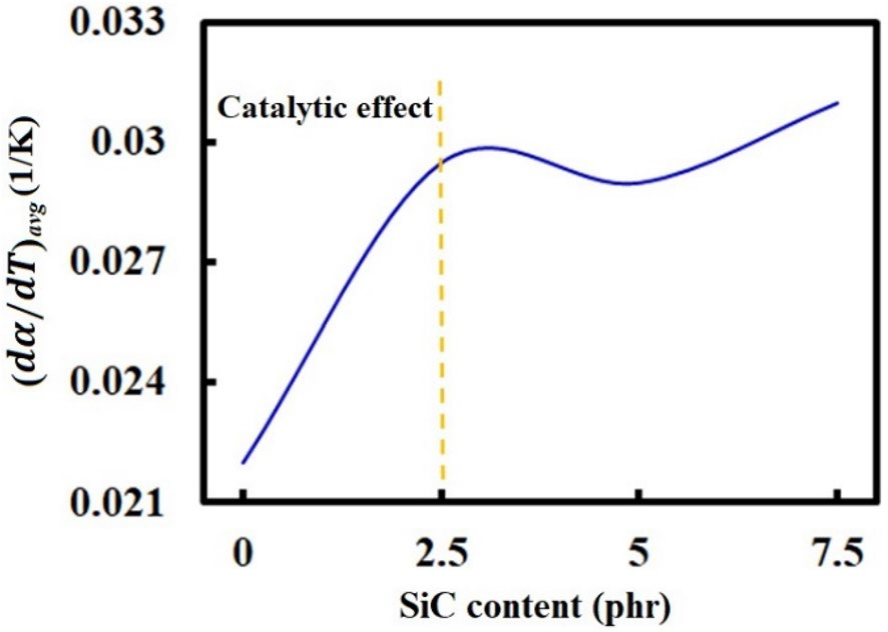
Effect of the SiC loading content on the mean of the maximum vulcanization rate (*dα/dT*)_*avg*_ of the SBR/BR-SiC compound*.*

Furthermore, the mean of the maximum vulcanization rate ($d\alpha /dT$)_*avg*_—an average over the maximum rates at different heating rates extracted from Fig. 7—versus the SiC content was calculated and reported in Fig. 8. As seen in the figure, the catalytic impact in SBR/BR-SiC with the SiC content of 2.5 phr is much further than the pure SBR/BR blend because of better heat distribution of SiC in the compound media. According to Fig. 8, increasing the filler loading content doesn’t almost influence the maximum rate of the vulcanization reaction significantly. Accordingly, the SiC content of 2.5 phr can be an excellent candidate to enhance the rate of the curing reaction.

### Thermodynamics of curing reaction of SBR/BR-SiC

In this research work, to measure the change in Gibbs free energy of the curing reaction of SBR/BR-SiC, the slope of the plot of *ln*(*k/T*) vs. 1000*/T* (see Eqs. 7 and 8) was obtained. According to the slopes, the variation of Gibbs free energy was calculated and listed in Table 4. Then, the equilibrium constant was calculated using the achieved $\Delta G$ s. The results of the equilibrium constant were also reported in Table 4.

**Table 4:** Thermodynamic characteristics of the vulcanization reaction of SBR/BR-SiC.

Sample code	$\beta$(K/min)	$\Delta G$(kJ/mol)	*lnK*
S0	10	544.58	− 138.78
	15	545.33	− 138.48
	20	543.69	− 137.65
	Mean	544.53 ± 0.67	− 138.30 ± 0.47
S1	10	412.05	− 105.84
	15	410.48	− 104.81
	20	410.96	− 104.58
	Mean	411.16 ± 0.65	− 105.07 ± 0.54
S2	10	254.82	− 66.608
	15	255.56	− 66.069
	20	255.27	− 65.375
	Mean	255.21 ± 0.30	− 66.017 ± 0.50
S3	10	223.07	− 59.197
	15	223.42	− 58.465
	20	224.10	− 57.937
	Mean	223.53 ± 0.42	− 58.533 ± 0.51

As seen in Table 4, the values of the obtained $\Delta G$ are far higher than zero, which means the curing reaction is forced. Moreover, according to the Le Chatelier–Braun rule^58^, the results of $\Delta G$ illustrate that the temperature increment leads to a shift in the reaction balance towards the reaction products. As exhibited in Table 4, the value of $\Delta G$ is decreased with increasing the SiC content, which means the vulcanization reaction is more easily proceeded in the compound with a higher amount of SiC filler. This achievement has conformity with the calculated activation energy (see Table 3). The achieved equilibrium constant also proves that the curing reaction is irreversible because its value is close to the limits of zero, indicating the reaction can be increasingly characterized as a one-way process.

## Conclusions

In this work, the cure characteristics, kinetics, and thermodynamics of the vulcanization reaction of the SBR/BR-SiC compound before and after the addition of high thermal conductive SiC were studied using non-isothermal DSC analysis. It was found that the presence of SiC in the compound media led to an increase in the onset temperature of the vulcanization peak. In comparison, the peak and end temperatures of the curing peak were reduced with the loading content of the SiC filler. Therefore, it can be concluded that the SiC particles accelerated the curing reaction enhancing the heat transfer into the compound. This phenomenon led to the increment of the enthalpy variation of the curing reaction into the compound, particularly at the SiC content of 5 phr. After that, the trend was changed because of the filler agglomeration. Moreover, the calculated activation energy based on the Kissinger method showed that the presence of SiC made the occurrence of vulcanization reaction easier. In addition, the autocatalytic model based on the Màlek method was applied to describe the kinetics of curing reaction in the compounds. The calculated kinetic parameters using the Sestàk–Berggren model by the Màlek method of the samples indicated that high thermal conductive filler within the SBR/BR-based compounds led to change in all the kinetic parameters. Also, the resultant data illustrated that the Sestàk–Berggren model still can describe the cure kinetics of the compounds even after the addition of SiC. Because the calculated reaction rates using the obtained kinetic model agree with the experimental values well. According to the results, it was found that the SiC filler has a catalytic effect on the vulcanization reaction of SBR/BR-SiC compound, particularly at 2.5 phr of the filler. This issue can help to reduce the required amount of energy consumption in the tire industry to cure the tire tread. Moreover, the kinetic parameters presented in this work can be used to obtain the curing kinetics of SBR/BR-SiC in the tire industry to achieve the curing degree at particular time and temperature. Furthermore, the calculated Gibbs free energy variation and equilibrium constant of the curing reaction of the compounds showed higher values than zero meaning the vulcanization reaction is forced. Accordingly, the curing reaction is an irreversible reaction that can be increasingly characterized as a one-way process. In general, the SBR/BR-SiC compound with 5 phr of SiC was found to be the best recipe due to the low peak and end temperatures, Gibbs free energy, and activation energy, as well as the highest enthalpy variation, which means the furthest amount of curing degree.

### Supplementary Information

Supplementary Information.

## Data Availability

The datasets used and/or analyzed during the current study are available from the corresponding author on reasonable request.
