# Supplementary material for: Catalytic effect of high thermal conductive SiC on the kinetics and thermodynamics of vulcanization reaction of SBR/BR-filled nano-SiC
Source: Sci Rep. 2023 Aug 30;13:14245. doi: 10.1038/s41598-023-41337-5 (PMC10469214; doi:10.1038/s41598-023-41337-5)
Supplement: Supplementary file 1 — Supplementary Information. [file 41598_2023_41337_MOESM1_ESM.docx]

Supporting Information to **“Catalytic Effect of High Thermal Conductive SiC on the Kinetics and Thermodynamics of Vulcanization Reaction of SBR/BR-Filled Nano-SiC”**

**Sajad Rasouli^1^, Amirreza Zabihi^2^, Mohammad Fasihi^3^^[[1]](#footnote-1)^**

^1^ School of Chemistry, Iran University of Science and Technology (IUST), P.O. Box 16844–13114, Tehran, Iran

^2^ Compounding Laboratory, Department of Technology, Kian Tire Manufacturing Company, Tehran, Iran

^3^ School of Chemical, Petroleum and Gas Engineering, Iran University of Science and Technology (IUST), P.O. Box 16844–13114, Tehran, Iran

This auxiliary information contains supportive evidences to strengthen the claims created in the main text.

**Table S1**. Characteristics of the vulcanization peak of the SBR/BR-filled SiC samples, according to the DSC curves presented in Figure 1.

| Sample code | $\beta$ ^a^ (K/min) | *T_o_* ^b^ (^o^C) | *T_e_* ^c^ (^o^C) | *T_p_* ^d^ (^o^C) | *∆H* ^e^ (J/g) |
| --- | --- | --- | --- | --- | --- |
| S0 | 10 | 140.1 | 249.5 | 198.8 | 16.25 |
|  | 15 | 163.2 | 261.8 | 200.5 | 16.76 |
|  | 20 | 171.1 | 248.6 | 201.9 | 15.35 |
| S1 | 10 | 169.8 | 239.2 | 195.1 | 17.68 |
|  | 15 | 162.3 | 243.1 | 197.9 | 13.83 |
|  | 20 | 172.4 | 244.7 | 199.5 | 19.74 |
| S2 | 10 | 170.3 | 235.0 | 187.0 | 26.25 |
|  | 15 | 170.6 | 240.9 | 192.1 | 13.47 |
|  | 20 | 177.6 | 245.5 | 196.5 | 13.17 |
| S3 | 10 | 172.1 | 232.6 | 180.1 | 12.84 |
|  | 15 | 168.2 | 233.0 | 186.5 | 8.54 |
|  | 20 | 177.3 | 243.2 | 192.1 | 35.87 |

^a^: Heating rate in the DSC analysis

^b^: Onset temperature of the curing peak

^c^: End temperature of the curing peak

^d^: Maximum temperature (or peak temperature) of the curing peak

^e^: Enthalpy variation of the curing peak


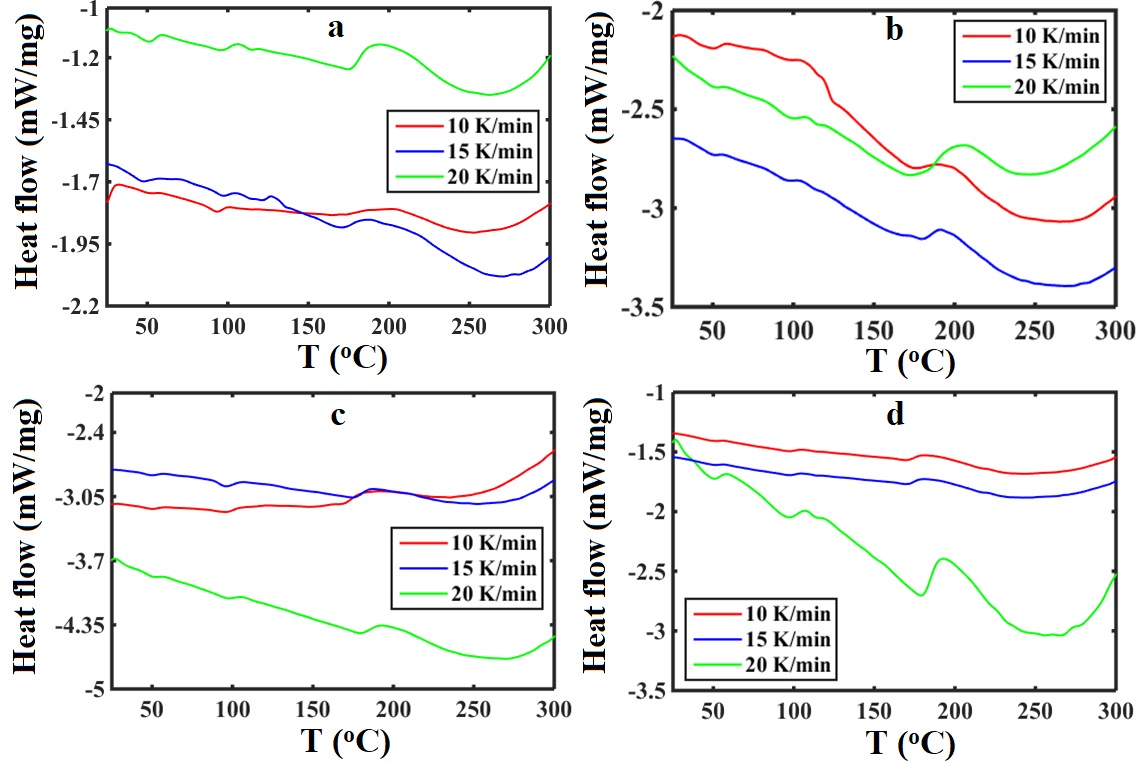


**Figure S1**. DSC curves of the SBR/BR-SiC samples with the filler contents of: (a) 0, (b) 2.5, (c) 5 and (d) 7.5 phr, at different heating rates of 10, 15 and 20 K/min.

1. mfasihi@iust.ac.ir [↑](#footnote-ref-1)
